# Morpho-Physiological and Proteomic Response of Bt-Cotton and Non-Bt Cotton to Drought Stress

**DOI:** 10.3389/fpls.2021.663576

**Published:** 2021-05-10

**Authors:** Swetha Sudha Nagamalla, Malini Devi Alaparthi, Sunitha Mellacheruvu, Ravindar Gundeti, Jana Priya Sony Earrawandla, Someswar Rao Sagurthi

**Affiliations:** Molecular Medicine Lab, Department of Genetics and Biotechnology, Osmania University, Hyderabad, India

**Keywords:** abiotic stress, MALDI-TOF/TOF MS, 2D difference gel electrophoresis, qRT-PCR, Cytoscape

## Abstract

Drought stress impacts cotton plant growth and productivity across countries. Plants can initiate morphological, cellular, and proteomic changes to adapt to unfavorable conditions. However, our knowledge of how cotton plants respond to drought stress at the proteome level is limited. Herein, we elucidated the molecular coordination underlining the drought tolerance of two inbred cotton varieties, *Bacillus thuringiensis*-cotton [Bt-cotton + Cry1 Ac gene and Cry 2 Ab gene; NCS BG II BT (BTCS/BTDS)] and Hybrid cotton variety [Non-Bt-cotton; (HCS/HDS)]. Our morphological observations and biochemical experiments showed a different tolerance level between two inbred lines to drought stress. Our proteomic analysis using 2D-DIGE revealed that the changes among them were not obviously in respect to their controls apart from under drought stress, illustrating the differential expression of 509 and 337 proteins in BTDS and HDS compared to their controls. Among these, we identified eight sets of differentially expressed proteins (DEPs) and characterized them using MALDI-TOF/TOF mass spectrometry. Furthermore, the quantitative real-time PCR analysis was carried out with the identified drought-related proteins and confirmed differential expressions. In silico analysis of DEPs using Cytoscape network finds ATPB, NAT9, ERD, LEA, and EMB2001 to be functionally correlative to various drought-responsive genes LEA, AP2/ERF, WRKY, and NAC. These proteins play a vital role in transcriptomic regulation under stress conditions. The higher drought response in Bt cotton (BTCS/BTDS) attributed to the overexpression of photosynthetic proteins enhanced lipid metabolism, increased cellular detoxification and activation chaperones, and reduced synthesis of unwanted proteins. Thus, the Bt variety had enhanced photosynthesis, elevated water retention potential, balanced leaf stomata ultrastructure, and substantially increased antioxidant activity than the Non-Bt cotton. Our results may aid breeders and provide further insights into developing new drought-tolerant and high-yielding cotton hybrid varieties.

## Graphical Abstract

Schematic diagram showing the differential responses of NCS BG II BT and Non BT varities to drought stress. NCS BG II BT variety showed better drought tolerance compared with Non BT plant variety.

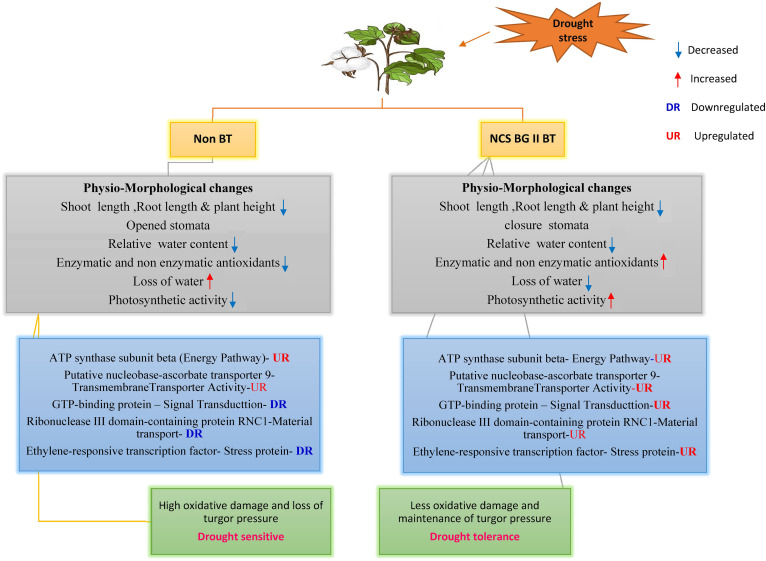

## Introduction

Cotton (*Gossypium hirsutum* L.) is the third-highest cultivated genetically modified (GM) commercial crop with an annual contribution of $500 billion to the world economy (Wilkins et al., 2000; Nakashima et al., 2012). Under stress conditions, the productivity of cotton is affected inimically (Selote and Khanna-Chopra, 2004). Drought is one of the abiotic stress factors, and it is anticipated that the proportion of droughty terrestrial areas could be double by the end of the 21st century. Under drought conditions, plants exhibit a varied response at whole-plant, cellular, and molecular levels. There is considerable genetic variations in cotton germplasm in response to drought (Babar et al., 2009). During chronic stress conditions, plants alter the expression of various proteins, and consequently, their biological functions are altered (Cohen et al., 2010; Kantar et al., 2011). At the cellular level, drought stress causes biomass accumulation, disruption of cellular homeostasis, and damage of chloroplast structure, constraining photosynthesis, facilitating reactive oxygen species (ROS) production, and increasing lipid peroxidation, leading to growth impairment (Zhang et al., 2017). At the whole-plant level, there is a decrease in photosynthesis and growth due to modifications in carbon and nitrogen metabolisms (Lawlor and Cornic, 2002). During evolution, at stress conditions, plants developed complex adaptive mechanisms, such as creating new signal transduction networks by transmitting stress signals to transcription factors (TFs) and elevating the levels of the phytohormones such as abscisic acid (ABA) (Zhu et al., 2017) in which ABA mediates stomatal regulation, the accumulation of non-enzymatic and enzymatic antioxidants, collection of osmoprotectants, and transcriptional activation of drought-responsive genes. Thus, drought tolerance of plants includes multigenic traits involving the interplay of many genes (Zou et al., 2010; Kim et al., 2012; Xu et al., 2012).

Plants efficiently use their components and energy to defend against various stresses. Earlier studies had shown that physiological processes are more prone to variable degrees of regulation under adverse conditions (Cramer et al., 2013^;^ Xie et al., 2016). An approach to stress tolerance by modifying TF levels has clear advantages over other strategies that focus on enhancing individual protein levels. Therefore, the differential expression of genes pertaining to TFs is a promising strategy for stress tolerance enhancement. A thorough understanding of drought tolerance mechanisms in cotton at the genome expression level has to be elucidated to develop drought-tolerant varieties. Proteome analysis provides crucial information related to ultimate executors in varied biological processes (Dov Greenbaum et al., 2003) and aids in characterizing the whole protein at the cellular or tissue level (Sobhanian et al., 2010). Two-dimensional polyacrylamide difference gel electrophoresis (2D-DIGE) is an effective technique for separating proteins (O’Farrell, 1975). Diverse factors can affect plant growth in stress and non-stress conditions. GM crop offers an unintended effect in the plant genome, resulting in the disruption and rearrangement of the genome and expression of new proteins. Thus, the evaluation of transgenic events and proteome response with induced conditions is essential (Conner and Jacobs, 1999; Gong and Wang, 2013).

In India, cotton cultivation was revolutionized after BT introduction. At present, only Bt transgenic hybrids or hybrid crops were grown mostly. As a result, post-Bt productivity was exaggerated from 303 kg/ha in 2001–2002 to 526 kg lint/ha in 2008–2009 (Venugopalan et al., 2009). Compared to the planet average, productivity levels per unit area are still low due to abiotic constraints.

This study evaluated and identified drought-responsive proteins in two GM cotton cultivars (NCS BG II BT containing Cry1 Ac gene and Cry 2 Ab gene and Non-Bt without Cry1 Ac gene and Cry 2 Ab gene). We have identified differentially expressed proteins (DEPs) using 2D-DIGE and matrix-assisted laser desorption ionization-time of flight/time-of-flight high-resolution tandem mass spectrometer (MALDI-TOF/TOF MS). Further confirmation of the DEPs carried out using quantitative real-time PCR (qRT-PCR) and these proteins was characterized by using different bioinformatic tools.

## Results

### Morphological and Physiological Changes in Plant Leaf and Variation in Growth

**FIGURE 1:**
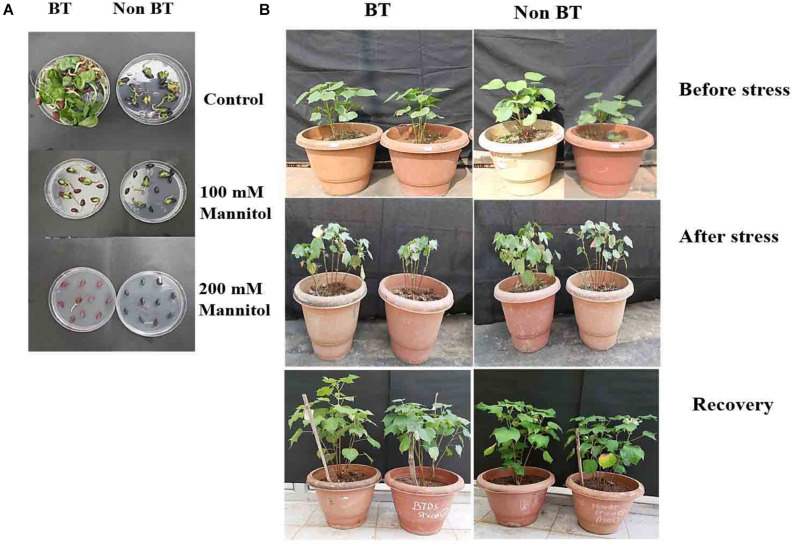
Performance of BT and Non-BT cotton plants against drought stress. **(A)** BT and Non-BT seed germination Control without mannitol, with 100 mM mannitol and with 200 mM mannitol. **(B)** BT and Non-BT cotton plants (60–65 days old) were subjected to drought (200 mM) for 15 days at 30°C. Later, the treated plants were allowed to grow under normal conditions for 20 days and later photographed.

We analyzed the photosynthetic efficiencies of BT and Non-Bt cotton plants on the 0, 4th, 8th, and 12th days under drought stress conditions. BT and Non-Bt cotton plants had shown different morphological changes in plant leaf and growth pattern development (Figure 1) and significant reduction in photosynthetic rate in both cotton leaves.

**FIGURE 2:**
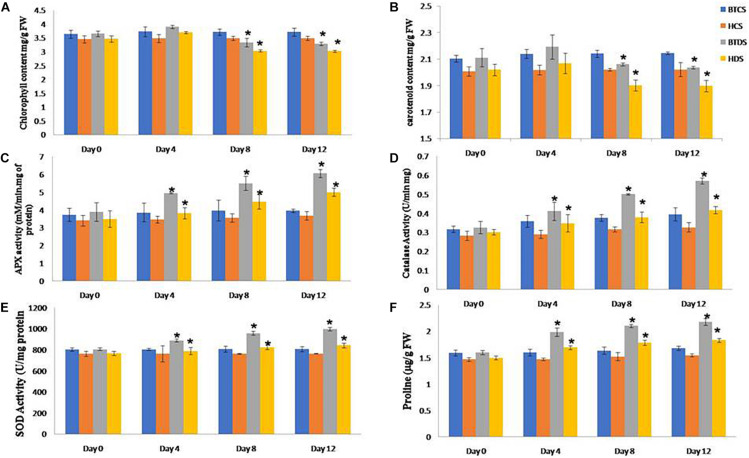
Biochemical characterization of BT and Non-BT cotton lines against drought stress condition. For analyzing the stress tolerance ability of Bt and Non-Bt cotton plants at the 1-month stage, drought stress (200 mM mannitol) was applied for 10 days at 30°C. Later, the treated plants were allowed to grow under normal conditions. After 0, 4, 8, and 12 days of mannitol stress, the samples were used for analysis. Data on **(A)** chlorophyll content, **(B)** carotenoid, **(C)** APX, **(D)** catalase, **(E)** SOD, and **(F)** proline were recorded and photographed. In each treatment, 10 seedlings of BT and Non-BT varieties were used. Bar represents mean and I represents SE from three independent experiments. ^∗^indicates significant differences in comparison with the C at *P* < 0.05, FW represents fresh weight.

The leaf relative water content (RWC) experiment shows that the leaf water retention capacity was higher in the Bt variety compared to Non-Bt under drought stress conditions. The Non-Bt cotton plant’s root and shoot length decreased by 28 and 17%, respectively, compared to Bt, when subjected to mannitol stress. The analysis specified Bt cotton was more tolerant than Non-Bt cotton to maintain root, shoot elongation, and plant growth during drought (Supplementary Figures 1, 2). On observation, it is noted that the chlorophyll and carotenoid content in both varieties were increased in stress conditions for the first 4 days and decreased in levels up to 10 and 42% in Bt and 13 and 47% in Non-Bt plants in the next 8–12 days (Figure 2).

**FIGURE 3:**
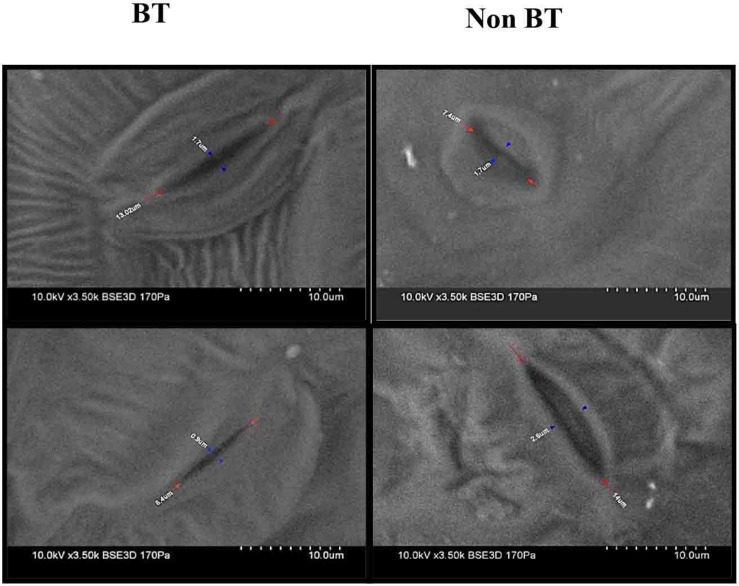
Observations of leaf stomata under a scanning electron microscope of BT and Non-BT cotton.

Scanning electron microscopy (SEM) studies on stomata revealed that opening was reduced from 1.7 to 0.9 μm in Bt leaves. Contrary to the Non-Bt stomatal opening, it was expanded to 1.7–2.6 μm, with the above observation showing that Non-Bt plants were more sensitive to drought stress than Bt plants (Figure 3).

### Antioxidant Assays in Bt and Non-Bt Ccotton Leaves Under Drought Stress Conditions

Under dehydration stress conditions, the activities of SOD, POD, CAT, and APX enzymes show dramatically higher antioxidant defense systems in Bt compared with Non-Bt. Similarly, the accumulation of proline content was also higher in Bt under drought stress. These results revealed that Bt has higher oxidative stress resistance compared to Non-Bt varieties by retaining ROS homeostasis via elevated antioxidant potential (Figure 2).

### Proteome Analysis in Both Varieties Under Drought Stress

**FIGURE 4:**
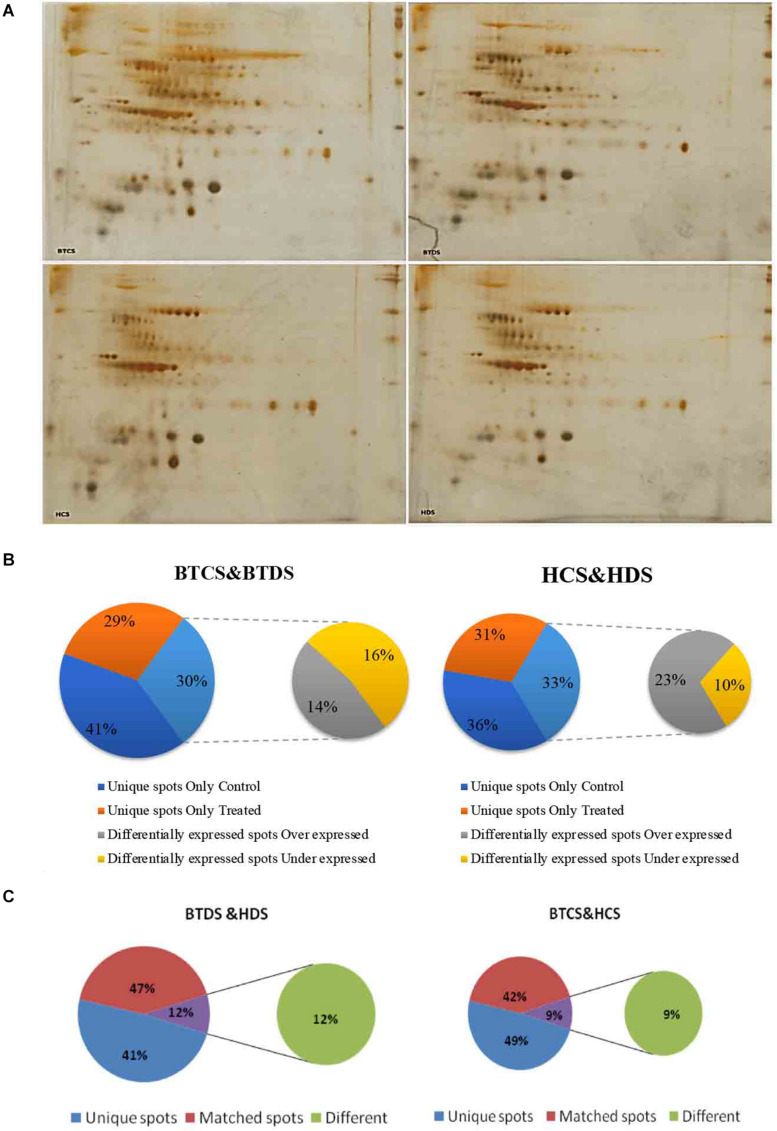
**(A)** 2-DE analysis of protein. Proteome maps of Intra-*hirsutum* Hybrid (*Gossypium hirsutum*) cotton varieties (BT hybrid—BTCS and BTDS and Non-Bt varieties—HCS and HDS). The numbered protein spots were identified by MALDI-TOF from BTCS and HCS grown in well-watered conditions, and BTDS and HDS grown under drought stress with 200 mM mannitol. **(B)** Venn diagrams of BTCS/BTDS and HCS/HDS. Venn diagrams representing the overlap of identified DEP spots, which were underexpressed and overexpressed. Unique spots in between groups such as BTCS and BTDS, and HCS and HDS. **(C)** Venn diagrams of BTCS/HDS and BTCS/HCS. Venn diagrams representing the overlap of identified DEP spots, which were unique spots, different spots, and matched spots in between groups such as BTDS and HDS, and BTCS and HCS.

To present additional conformational assessment on biosafety of Bt and Non-Bt cotton, we applied a proteomics-based approach by investigating the DEPs between Bt cotton leaves and their Non-Bt counterparts under induced drought conditions. Proteins were extracted and quantified from both controls and stressed plants, further proceeding with 2-DE electrophoresis (Figure 4A). We observed a marked variation in expression profiles. Supplementary Tables 1A–C show the total number of spots, unique, overexpressed, and underexpressed in BTCS, BTDS, HCS, and HDS. Venn diagrams in Figures 4B,C represent the percentage of the corresponding data. Only the significantly abundant DEPs with a fold change of 1.5 (confidence above 95%, *P* < 0.05) were selected for further analysis.

### Analysis of DEPs

We identified eight DEPs in Bt and Non-Bt plants by peptide mass fingerprinting (PMF) and MALDI-TOF/TOF-MS after careful scrutinization of 2D-DIGE in both cotton varieties. Protein spots were identified based on molecular weights using MASCOT software. HDS-Spot (493) is ATP synthase subunit beta (ATPB) with accession number YP_913194 and is involved in hydrogen ion transport in ATP synthesis. Spot 434 in HCS is Putative nucleobase-ascorbate transporter 9 (NAT9), with accession number Q3E956 and aids in ion transportation. BTDS-Spot (730) is Protein reticulata-related 6 (RER6), with accession number XP_002876351, and supports leaf development. Spot (852) is GTP-binding protein (GTPB) with accession number XP_002878640 and has multiple functions, such as ligand GTP binding, metal binding, and nucleotide binding. Spot (844) is ethylene-responsive transcription factor RAP2-3-like (ERF) with accession number NP_001314374 and is capable of DNA binding and transcription regulation. HDS-Spot (389) is also ATPB, involved in ATP synthesis and energy metabolism. Spot (406) is a Ribonuclease III domain-containing protein (RNC1) with accession number XP_015648830 and assists in ribonucleoprotein formation and RNA binding material transport. Spot (164) is Ribulose bisphosphate carboxylase/oxygenaseactivase B (RuBisCO) with accession number Q7X9A0 and is capable of ATP-dependent carboxylation (Table 1).

**TABLE 1:** Characterization of differentially expressed proteins.

**S.no**	**Spot ID**	**MASCOT score**	**Sequence coverage (%)**	**MW/pI**	**Protein name**	**Accession number**	**Organism**	**Function**
1	HDS 406	70	35	60/5.69	Ribonuclease III domain-containing protein RNC1	XP_015648830	*Oryza sativa* subsp. japonica (Rice)	Ribonucleo protein, RNA-binding- Material transport and
2	HDS 412	164	29	56/5.27	Ribulose bisphosphate carboxylase/oxygenase activase B	Q7X9A0	Larreatridentata (Creosote bush) (Zygophy llumtridentatum)	ATP-dependent carboxylation
3	HCS 391	748	68	79/5.39	ATP synthase subunit beta	YP_913194	*Gossypium barbadense* (Sea-island cotton) (Egyptian cotton)	ATP synthesis, Hydrogen ion transport, Ion transport, Transport
4	HDS 389	564	58	72/5.43	ATP synthase subunit beta	YP_913194	*Gossypium barbadense Gossypium hirsutum* (Upland cotton)	ATP synthesis- Energy metabolism
5	BTDS 844	321	35	39/4.94	Ethylene-responsive transcription factor RAP2-3-like	NP_001314374	*Gossypium hirsutum*	DNA binding and transcription regulation
6	HCS 434	66	29	46/5.57	Putative nucleobase-ascorbate transporter 9	Q3E956	Arabidopsis thaliana (Mouse-ear cress)	Transport
7	BTDS 872	113	47	34/4.27	GTP-binding protein	XP_002878640	Arabidopsis thaliana (Mouse-ear cress)	Ligand GTP-binding, Magnesium, Metal-binding, Nucleotide-binding
8	BTDS 730	62	27	90/4.6	Protein reticulata-related 6,	XP_002876351	Arabidopsis thaliana (Mouse-ear cress)	Leaf development

### Subcellular Localization Studies

**FIGURE 5:**
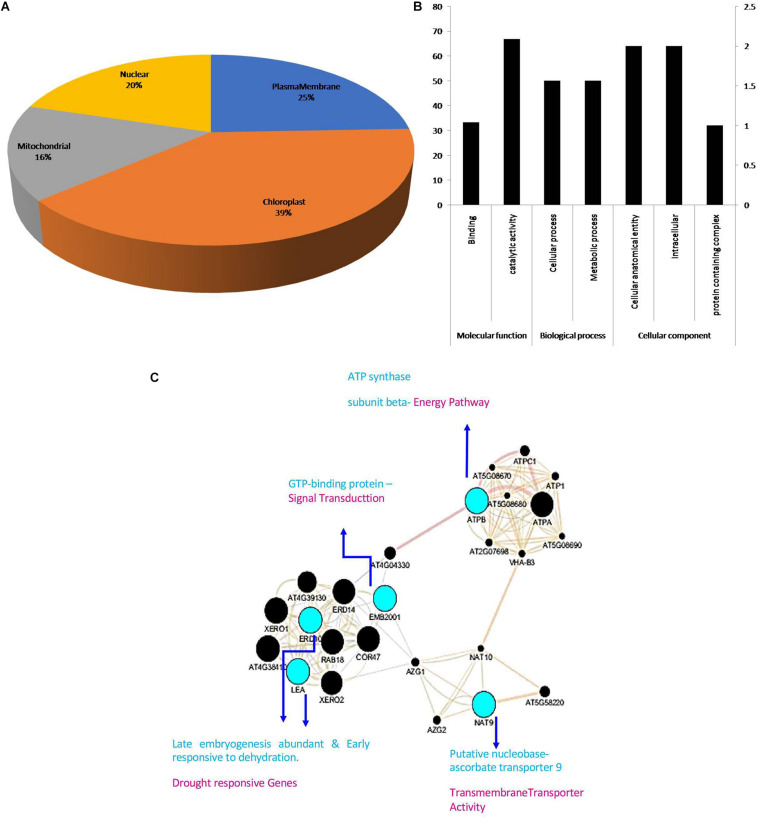
**(A)** The subcellular locations of the identified proteins. **(B)** GO classification of the identified DEPs. To reveal the functions of the identified eight DEPs between Bt and Non-Bt, GO functional analysis was carried out using Panther classification system software. The identified eight DEPs were classified into three main categories including cellular component, biological process, and molecular function with seven subgroups. The number of genes signifies that of proteins with GO annotations. **(C)** Interactions of these differentially accumulated proteins are extracted using the app GeneMANIA from Cytoscape.

The subcellular localization of the identified proteins affirms the presence of four proteins in chloroplast, three in plasma membrane, and one each in nucleus and mitochondria (Figure 5A). The large numbers of proteins present in chloroplast and plasma membrane suggested that DEPs were involved in carbohydrate transport and metabolism.

### GO Analysis of Identified Proteins Using PANTHER

To reveal the functional variations of DEPs between Bt and Non-Bt, PANTHER classification system GO analysis was performed to validate the cellular component, biological process, and molecular function. Depending on functional annotation, eight DEPs were classified into three major groups having seven subgroups. Two subclasses were assigned through the molecular function ontology. The vast segment was catalytic activity (GO: 0003824) comprising two proteins and one protein with binding function (GO: 0005488). In the biological process category, two subgroups were allocated. The major part consisting of one protein was related to cellular process (GO: 0009987) pursued by metabolic process (GO: 0008152), involving one protein. At the GO-cellular level, the largest part including two proteins was in the cellular anatomical entity (GO: 0110165); another protein is in the protein-containing complex (GO: 0032991) and two proteins arise in the intracellular region (GO: 0005622) (Figure 5B).

### Interaction of DEPs

The GeneMANIA database is used to identify the interaction of the identified DEPs, which utilizes co-expression and experiment conditions for network construction. Cytoscape (version 3.7.1) software was used to construct and to visualize the network of interactions between proteins. ATPB, NAT9, RER6, GTPB, ERF, RNC1, and RuBisCO proteins were used to investigate these interactions with the above software in our present study. In this network analysis, three major clusters of protein interactions were interconnected: blue indicates identified proteins, green shows other proteins, red indicates co-expression, orange denotes predicted functions, and light green signifies shared protein domain (Figure 5C).

### Expression Studies

**FIGURE 6:**
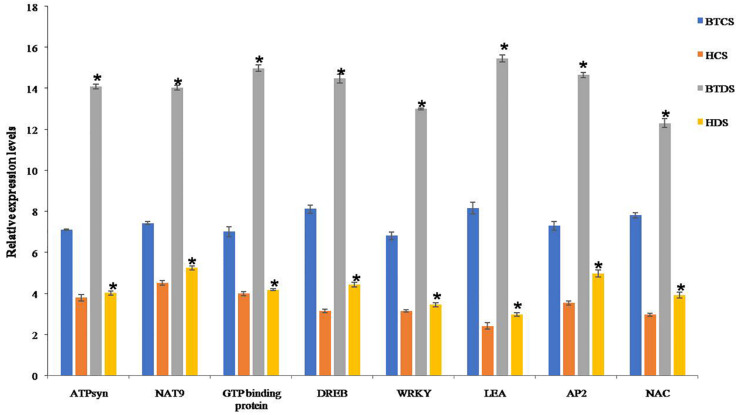
Real-time PCR analysis of ATP Syn, NAT9, GTP-binding protein, DREB, WRKY, LEA, AP2/ERF, and NAC genes in BT and Non-BT cotton variety plants under drought stress conditions. Relative transcript levels of genes in BT and Non-BT cotton plants subjected to 2000 mM mannitol stressed condition were analyzed using real-time PCR. Actin gene was used as a reference. Bar represents mean, and I represents SE from three independent experiments. Asterisk indicates significant differences in comparison with Non-BT at *P* < 0.05.

Finally, genes responsible for drought were validated by qRT-PCR. To see the concordance between proteins and mRNA transcription patterns, we selected interconnected proteins NAT9, ATPB, EMB2001, DREB-ERD, and drought-responsive genes LEA, AP2/ERF, WRKY, and NAC. The change in mRNA expression of these genes was comparable in controls, and expression levels were increased in Bt compared to Non-Bt in drought stress conditions (Figure 6).

## Discussion

Our results revealed that both BTDS and HDS major morpho-physio alterations such as leaf RWC, ROS, amino acids, and protein levels have been recorded under drought conditions relative to controls (BTCS and HCS). Wang et al. (2003) and Hasanuzzaman et al. (2013) have also stated that these morpho-physio alterations under drought stress severely inhibit its growth as well as development. Morphological changes include a relative decrease of root length, shoot length, and leaf surface area recorded under drought conditions. Similar experiments have shown that a reduction in shoot and root length is specifically linked to reduced photosynthesis (Bhatt and Rao, 2005; Koroleva et al., 2005; Ashraf and Foolad, 2007; Shao et al., 2008), loss of water, loss of turgor pressure, and reduction in chlorophyll and carotenoid content under dry conditions in different plant varieties. Likewise, chlorophyll and carotenoid content were also reduced in Bt plant varieties compared to their controls. Previous studies reported a decrease in chlorophyll and carotenoid content, ultimately reducing the photosynthetic activity (Ali et al., 2017; Mahmood et al., 2017).

Water levels in leaf is a good marker of drought tolerance; it is measured as a function of leaf RWC, which is very strongly related to plant water potential (Nakano and Asada, 1981; Sánchez-Blanco et al., 2002). The leaf RWC was influenced by several leaf physiological characteristics. When subjected to drought stress, RWC was decreased by less than 80%, which ultimately affects photosynthesis up to 50% (Gujarathi et al., 1984; Parida et al., 2007; Ullah et al., 2012). We observed persistent high water levels in Bt cotton variety compared to Non-Bt cotton in both conditions.

Under tropical climatic conditions, stomatal closure increases to prevent dehydration from leaves. Recurring drought conditions increase the sensitivity of stomatal closure to prevent water evaporation and inhibit the diffusion of CO_2_ to the carboxylation site, as well as decrease photosynthetic uptake (Zhang et al., 2005). We observed a remarkable decrease in the opening of stomata in BTDS compared to BTCS. However, in HDS, the stomatal opening was wider than that in HCS, which is in contrast to Bt. Our data suggest that Bt plant leaves show drought tolerance than Non-Bt (Aasamaa and Sõber, 2011).

ROS are associated with antioxidant proteins during plant growth under stress (Serrato et al., 2004). The exposure of plants to drought stress leads to increased oxidative stress due to enhanced accumulation of ROS. As a result, antioxidant enzyme activity was increased to conquer the oxidative damage of biomolecules (Becana et al., 2000; Shah et al., 2001). Our findings showed insignificant disparity between Bt and Non-Bt in normal conditions. However, the antioxidant enzyme concentration was dramatically higher in Bt plants; it may have contributed to maintaining photosynthetic activity and membrane stability under drought stress. In contrast, Non-Bt plants showed a decreased antioxidant enzyme activity and increased cell death. With these observations, Bt plants exhibit drought tolerance compared to Non-Bt plants.

Usually, arginine, proline, asparagine, and amides, like free amino acids, were stimulated by drought stress (Lubbers et al., 2007). Under stress conditions, proline plays a protective role in support of osmotic adjustment and in the recovery of the plant from cellular dehydration (Szabados and Savouré, 2010; Kaur and Asthir, 2015; Per et al., 2018). Previous studies suggested that increased proline content enhances stress tolerance (Sánchez et al., 1998; Hare et al., 1999; Szabados and Savouré, 2010; Man et al., 2011; Sultan et al., 2012) and mentioned higher proline levels in drought-tolerant specific species of cotton, tall fescue, and wheat. Our results also consistently exhibited higher accumulation of proline levels in Bt than in Non-Bt, under stress conditions.

Theoretically, when living surroundings of cells are distorted, the proteome composition of the cell consequently changes. These alterations are essential for cell to acclimatize in varied environments (Kottapalli et al., 2008). Proteome analysis by 2DGE and PMF-based MALDI-TOF/TOF-MS reveals that the changes among them were not obviously in respect to their controls (Ren et al., 2009; Brandão et al., 2010; Gong et al., 2012; Tan et al., 2016), yet photosynthetic proteins greatly influenced both varieties to an analogous degree under drought conditions. In comparison with their Non-Bt, approximately eight DEPs were found in the Bt cotton leaves, which displayed differential expression in our plants and were involved in essential plant growth mechanisms, photorespiration, cell defense, and metabolism of amino acids.

Proteins RNC1 and RuBisCO, massive monetary unit binding proteins, play a crucial role in photosynthesis. RuBisCO (982, 410, and 412) is an essential protein concerned in photosynthetic carbon assimilation in the reductive pentose phosphate cycle and modulates the link between photosynthesis. The primary step in photorespiration is the oxidation of RuBP (Ribulose 1,5-bisphosphate), which is catalyzed by RuBisCO to generate one molecule of phosphoglyceric acid and phosphoric acid. Therefore, both photosynthesis and photorespiration are regulated by RuBisCO; during these processes, a significant amount of energy is altered to heat, which means crop yields are limited (Law et al., 2001; Spreitzer, 2003). Increased protein expression was recorded in both plants, although when we compare Non-Bt lines to Bt lines, the former showed decreased expression levels in drought conditions. Other researchers showed RuBisCO protein to be increased at both protein and transcriptional expression in the transgenic cotton lines (Aranjuelo et al., 2011; Wang et al., 2011, 2015, 2016; Deeba et al., 2012; Chen et al., 2019).

Proteins concerned in energy metabolism provide energy for transportation and metabolism. In this study, ATPB (spots 389, 391, and 493) proteins were upregulated in Bt and Non-Bt hybrid. The upregulated ATP synthase beta monetary unit 1 assists in the degeneration of macromolecules in chloroplasts and interferes in photosynthesis in drought stress. The synthesized ATP provides energy for transport and signal transduction to take care of the plant’s growth at the identical time. Some studies suggested that during drought stress, expression of ATP synthase beta subunit was decreased (Tezara et al., 1999; Valero-Galván et al., 2013; Cao et al., 2017). Conversely, Kottapalli and Zhou’s studies show that ATP synthase beta subunit expression was increased with drought stress, stating that generation of intolerant protein peanut genotypes may minimize water stress by increasing ATP to meet energy demand during stress, and these results were comparable with our results (Kottapalli et al., 2008; Zhou et al., 2015). Our results show the overexpression ATP synthase gene in both Bt and Non-Bt hybrid cotton compared to controls. Similar results were shown in *Arabidopsis* and assumed that the ATP synthase gene’s overexpression imparts greater tolerance in drought (Zhang et al., 2008).

The expression of ERFs (2,540, 844, and 466) affects overall plant growth and development. In our present study, ERF-RAP2-3-like-ERD were upregulated in Bt and downregulated in Non-Bt plants after drought stress. Because of biotic or abiotic stresses, the ERFs are diversely expressed in various plants. Previous studies show that under drought stress, overexpression of ERF can improve plant tolerance to drought in various plants such as *Gossypium herbaceum*, sugarcane SodERF3, tomato TERF1, *Brassica rapa* BrERF4, and Arabidopsis (Qiao et al., 2008; Jin et al., 2009), and with these results, we observed that Bt plants exhibit higher drought tolerance than Non-Bt plants (Seo et al., 2010).

In this study, spots 2,509, 810, and 434 purported that NAT9 proteins are essential for the transit of nucleobases. Under stress, these proteins played a part in diverse plant growth and developmental processes (Chai et al., 2018). NAT9 was involved in material transport, nutrition, cell signaling, stress responses, and cell homeostasis. These proteins were upregulated in both plants in response to drought stress. Our data are supported by Sun and Tingting’s studies that show that the expressions of NAT proteins were increased in apple plant by drought and salt stress (Lloyd, 1989; Jiang et al., 2007; Yang et al., 2008; Sun et al., 2016).

Rab proteins (2,547 and 872) belong to Ras superfamily of guanosine triphosphate (GTP)-binding proteins involved in swapping between “active” and “inactive” states at the molecular level. They are involved in different cell functions like cell proliferation, signal transduction, cytoskeletal organization, gene expression, and intracellular membrane trafficking. Tiny GTPBs have two interchangeable states, the GDP-bound inactive type and the GTP-bound active form (Bourne et al., 1990). On stimulation, GTP binds to the inactive form of G protein-coupled receptor by dissociating GDP, resulting in downstream conformational changes at the effector-binding region (Agarwal et al., 2009). In the abiotic stress response, GTPBs is considered critical regulatory factors in diverse cellular processes (Kim et al., 2012). Our results demonstrate that the levels of GTPBs were upregulated in Bt compared with their controls and the expression levels also increased in Bt but decreased in Non-Bt in drought stress plants. The results were consistent in a similar approach to the proteomic response of maize plants to dehydration stress (Shao et al., 2015). Thus, during drought conditions, upregulation of GTPB may act as a positive signal.

RER6 (730 and 374) was concerned with leaf development, which needs the advanced coordination of genetic and metabolic factors for differentiation of tissue varieties with divergent functions. The known protein performance patterns propose the existence of defense mechanisms operational throughout the early stage of infection that differed in each verity. In drought conditions, morphologically, leaf size is decreased to reduce water loss during respiration; at the physiological level, RER6 expression subsides. Accordingly, reticulate formation in leaves is also deceased. Our results also support the same; RER6 expression was decreased in both plant varieties (Pérez-Pérez et al., 2013).

All the eight DEPs, namely, RuBisCO, RER6, RNC1, ATPB, NAT9, ERF-RAP2-3-like—ERD, and GTPB—EMB2001, were used for bioinformatics analysis. Cytoscape-GeneMANIA networks find the interrelated genes associated with a collection of input genes, employing a huge set of functional data. Gene and protein interaction pathways, localization, co-expression, and protein domain resemblance conditions were used to construct the network (Mostafavi et al., 2008).

ATPB, NAT9, ERD, LEA, and EMB2001 were allied networks and other protein networks that are structurally and functionally correlative. These data revealed that upon upregulation, some DEPs were directly correlated with dehydration-responsive proteins, which were concerned with carbon fixation in photosynthesis organisms and photosynthesis (Limin Wang et al., 2015). After observing these interactions, we confirmed the concordance between protein and gene interactions with RT-PCR analysis.

Expression analysis was performed with NAT9, atpB, EMB2001, DREB–ERD, and some interconnected drought-responsive genes LEA, AP2/ERF, WRKY, and NAC. Under stress conditions, the expression of DREB, WRKY, AP2, and NAC was upregulated in Bt plants. These TFs bind to promoters of downstream genes that were induced during drought stress, thereby activating overexpression of drought-related genes and directly controlling the expression of the allied genes by acting as molecular switches. TFs regulate the gene expression after binding with cis elements located on the promoter region. In plants, a large percentage of genes in the genome potentially encode TFs (Ullah et al., 2012).

DREB proteins interact with stress-responsive genes at cis-acting elements, thereby controlling their expression (Dubouzet et al., 2003). Similarly, WRKY TFs are implicated in different biological processes in plant development. NAC TFs are engaged in several processes, like development of flower, secondary wall, shoot apical meristem, aging of leaves, and cell division in both biotic and abiotic stresses (Olsen et al., 2005; Tran et al., 2010; Nakashima et al., 2012; Banerjee and Roychoudhury, 2015). In general, late embryogenesis proteins were induced by ABA treatment, and they were also found to confer good tolerance to stress conditions. AP2/EREBP genes play an essential role in plant growth and development under stress responses (Liu and Zhang, 2017). They upregulated the expression levels of AP2 in BTCS and BTDS compared with HCS and HDS plants.

Between living cells, some enzyme expression levels were elevated, rendering imbalance, which can result in the various drought tolerance skills of drought-tolerant and drought-sensitive varieties. Consequently, some enzymes ought to be downregulated to retain balance. In line with the proteomic study of drought-stressed cotton leaves, drought stress will have a bearing on the leaf proteome of cotton with varied drought tolerance abilities to numerous degrees. The identified drought-sensitive TFs are less than reported by others in different plants (Seki et al., 2007). In cotton, several TFs, such as DREB, ERF, ATPB, and NAT9, that play a crucial role in drought tolerance were identified in this study (Qiao et al., 2008). In our findings, some DEPs were directly interconnected with dehydration-responsive element-binding factors and were upregulated in Bt compared to Non-Bt.

The underwater deficit, crop varieties, and transgenic and environmental conditions may also affect plants’ gene expression. Some of the drought-induced proteins had a massive significance for the identification of drought tolerance in cotton varieties. Depending on the results, we revealed the expression of few proteins that were modified after stress treatment, like RuBisCO large subunit-binding proteins α and β subunits, ATPB, ERF, and NAT9.

The overall results demonstrate that Bt line expression has intense effects on stress tolerance of cotton plants. Finally, we possibly assume that these proteins may be used as protein markers to identify drought tolerance in cotton varieties. The relative expression profiles of Bt and Non-Bt are useful to consolidate our knowledge in understanding the mechanism of cotton plant in drought stress. Significant metabolic changes of cotton plant leaves are also concerned with drought tolerance and adaptation and supply new insights into the plant drought response’s proteomic mechanisms.

## Conclusion

This study provides a relative analysis of morphological, physiological, and differential protein expression under control and drought conditions in leaves of commercial cotton varieties (Bt hybrid—BTCS and BTDS and Non-Bt varieties—HCS and HDS). Our morphological observations and biochemical experiments showed a different tolerance level between two inbred lines to drought stress. These protein conserved patterns have differed in both types throughout the early stages of stress. Leaf proteins concerned with various functions like ATPB (YP_913194) metabolism and hydrogen ion transport in ATP synthesis and NAT9 (Q3E956) aiding in cell signaling, nutrition, material transport, stress responses, and cell homeostasis had shown differential expression. Other proteins, such as RER6 (XP_002876351) that supports leaf development and GTPB (XP_002878640), have multiple functions, namely, ligand GTP binding, metal binding, and nucleotide binding; ERF (NP_001314374) DNA binding and transcription regulation affect plant growth and development. Whereas RNC1 (XP_015648830) and RuBisCO (Q7X9A0) were predicted to play a role in photosynthesis and photorespiration, RuBisCO was also involved in ATP-dependent carboxylation. Proteome analysis reveals that the changes among them were not obviously in respect to their controls, yet photosynthetic proteins greatly influenced both varieties to an analogous degree under drought conditions. In our findings, some DEPs were directly interconnected with dehydration-responsive element-binding factors (TFs) and were bound to promoters of downstream genes that are induced during drought stress, thereby activating overexpression of drought-related genes. Thus, the results recommended that the inserted Cry1 Ac gene and Cry 2 Ab gene in Bt plant directly or indirectly effect the plant growth response to induced drought. Therefore, the identified genes responsible for drought tolerance may further assist cotton breeders to grow new drought-tolerant and high-yielding cotton verities.

## Materials and Methods

### Plant Materials

The experimental cotton (*G. hirsutum* L.) intra-*hirsutum* hybrid-NCS BG II BT+ Cry1 Ac gene and Cry 2 Ab gene (BTCS/BTDS) and their Non-Bt-Cry1 Ac gene and Cry 2 Ab gene varieties (HCS/HDS) were cleaned, sterilized, and immersed in water for a day at 30°C and then transferred to a petri dish for germination in a moist 28/25°C as day and night temperature, 12 h of light and dark periods alternatively with a relative humidity of 80%. After 3 days, germinated seeds were transferred into pots. For the drought stress treatment, 200 mM mannitol was applied for 10 days at 30°C, followed by growing plants at normal conditions (Możdżeń et al., 2015). The leaves were gathered and instantly frozen in liquid nitrogen after stress handling, lyophilized, ground to a fine powder, and preserved in humidity-proof containers at −80°C before analysis. There were three replications of each treatment, with 12 plants per replicate.

### Evaluation of Bt and Non-Bt Cotton Varieties for Drought Stress

Drought stress influences various biochemical, morphological, and physiological changes and leads to drastic effects on plant growth. Measurement of plant chlorophyll and carotenoid content, stomatal conductance, RWC, catalase, superoxide dismutase (SOD) activity, ascorbate peroxidase (APX) activity, and proline content can be used for identification of drought resistance of plants. Bt and Non-Bt cotton plant leaves were used for analysis of stress tolerance ability.

The data on biomass, survival rate, root length, and shoot length were recorded and photographed. All the stress experiments were repeated at least three times.

### Relative Water Content

To determine the fresh weight of leaves, they were harvested and recorded immediately. Besides, the leaves were dried at 80°C for three consecutive days in an oven to obtain dry weight.

The following formula (Barrs and Weatherley, 1962) was used to calculate leaf RWC:

RWC=(freshweight-dryweight)/(turgid⁢weight-dry⁢weight)× 100

### Observation of Stomata in the Leaf

Stomatal regulation shows a crucial role in protecting the plants from oxidative stress caused by drought stress. After drought stress, the plant leaves were collected and used for analysis. Bare leaves were used for detection of stomatal aperture and density using an SEM-S3400 (Hitachi, Tokyo, Japan) and analyzed by Image J software (National Institutes of Health, Bethesda, MD, United States).

### Chlorophyll and Carotenoid Content

Chlorophyll plays a vital role in photosynthesis; the photosynthetic rate can be estimated by measuring plant tissue’s chlorophyll content. One hundred milligrams of plant material was grounded in a pre-chilled mortar and pestle using 5 ml of chilled acetone. Homogenate was centrifuged at 10,000 rpm for 10 min at 4°C. The supernatant was collected and absorbance was measured at 470 and 750 nm for carotenoids and at 640 and 663 nm for chlorophyll content (Lichtenthaler and Wellburn, 1983).

### Catalase Activity

Catalase (CAT) activity was measured by using molar extinction coefficient 36 × 10^3^ mm^–1^ m^–1^; the decreased absorbance was measured at 240 nm and 30°C. Fifty microliters of enzymatic extract was added to 20 mM H_2_O_2_ and 50 mM potassium phosphate buffer at pH 7.0. The value was expressed as μmol H_2_O_2_ oxidized g^–1^ FW min^–1^ (Haake et al., 2002).

### SOD Activity

The photochemical inhibition of reduced nitro blue tetrazolium (NBT) was measured at 560 nm to determine SOD activity. The reaction mixture includes 50 mM NBT, 13 mM methionine, 1.3 M riboflavin, 75 mM EDTA, 50 mM phosphate buffer, and 50 μl of enzyme extract. The reaction was initiated by placing the sample under a 30-W fluorescent lamp, and the lamp was turned off to terminate the reaction. The determined SOD activity was given as SOD IU min^–1^ mg^–1^ protein (van Rossum and van der Plas, 1997).

### APX Activity

APX activity was measured using Nakano K method. Two milliliters of total reaction mixture contains 0.05 M potassium phosphate buffer (pH 7), 0.2 mM EDTA, 0.5 mM ascorbic acid, and 0.25 mM H_2_O_2_. The reaction was initiated with the addition of 0.1 ml of plant extract at 25°C. The decrease in absorbance was recorded at 290 nm for 1 min, and an extinction coefficient of 2.8 mM cm^–1^ was used to calculate the amount of ascorbate oxidized (Nakano and Asada, 1981).

### Proline Content

Proline content was measured in leaf, with minor modifications to Bates protocol and expressed as micrograms per gram per unit fresh weight (Bates et al., 1973). Approximately 1 g of plant samples was grounded with 3% (w/v) aqueous sulfosalicylic acid solution and centrifuged at 2,500 *g* for 30 min. Glacial acetic acid and acid–ninhydrin reagent were mixed in 1:1 ratio to the supernatant, and the mixture was boiled for 1 h. To the above mixture, 4 ml of toluene was added and centrifuged at 3000 rpm for 10 min. Proline content is determined by measuring the absorbance at 520 nm. L-proline was used as a standard to analyze the results further.

### Proteomic Analysis of Non-Bt and Bt Cotton Varieties

#### Protein Extraction

For protein extraction, 1 g of plant leaf sample was used and ground using liquid nitrogen. Metabolite Extraction Buffer is added and homogenized for 5 min and centrifuged at 10,000 rpm for 10 min at 4°C. Five volumes of SDS buffer (sodium dodecyl sulfate) with protease inhibitor were added further and incubated for 1 h at RT on a shaker to the pellet and centrifuged at 10,000 rpm for 15 min at 4°C. To the supernatant, equal volumes of Tris-buffered Phenol were added. After 30 min rocking, the sample was centrifuged at 10,000 rpm for 30 min at 4°C. Six volumes of 100 mM ammonium acetate/methanol solution were added and incubated overnight at −20°C to the lower phenol layer. The next day, the samples were centrifuged at 10,000 rpm for 15 min at 4°C. The pellet was washed with pre-chilled acetone and centrifuged at 10,000 rpm for 15 min at 4°C. The pellet was air-dried and dissolved in rehydration buffer. Sample proteins are extracted and quantified, and the sample concentration and the protein profile were checked using SDS-PAGE.

### Two-Dimensional Difference Gel Electrophoresis

Three hundred microliters of each sample (nearly, 300 μg of protein samples in rehydration buffer containing Urea, CHAPS, Dithiothreitol, and Biolyte) was loaded onto the IEF strip 3–10 pH linear, 18 cm, by rehydration in the Iso-Electric Focusing (IEF) tray. The gels were overlaid with mineral oil before placing in the IEF cell. The voltage was ramped rapidly from 250 to 10,000 V and run till 70,000 Vh is reached. After the IEF run, equilibration of strips was done in equilibration buffer (composed of urea, Tris–HCl, pH 8.8, SDS, and Glycerol) for 1 h each, followed by the treatment of strips with EB-1 (Equilibration Buffer + Dithiothreitol) for 1 h and subsequently treated with EB-2 (E Equilibration Buffer + Iodoacetamide) for 45 min and then placed on a 10% SDS-PAGE large format gel for running the second dimension in EttanDalt electrophoresis unit (GE).

Fixation was done overnight by using 50% methanol and 10% acetic acid. Excess of fixing reagent was removed by two to three water washes. After 2-h incubation in 0.02% sodium thiosulfate solution, the strip was treated with 0.2% silver nitrate solution for 1 h.

The gels were finally developed in 2% sodium carbonate solution after washing thoroughly with water and strips were stored in 10% acetic acid. The gels were scanned using the EPSON Expression 11,000XL. Protein spot expression analysis was performed using Image Master 2D Platinum 7 software. At least three biological replicates per sample was examined (Wang et al., 2007).

### Image Analysis

To identify the total number of spots and differentially expressed spots between two samples on 2D gels, Image Master 2D Platinum (GE healthcare, version 7.0.6.) was used. The software will automatically match all the gels’ spots and assign match ids for the matched spots in the gels and spot ids to all the spots in the gel set. Annotations were created indicating the pI and molecular weight for a few spots on the matched gels, and the software will annotate all the remaining spots. It has identified DEP spots from the control and treatment samples. The spot percentage of volumes is taken into consideration to predict the fold change of differentially expressed spots between the gels.

Fold change was calculated as the ratio of percentage volumes of treated spots to percentage of volumes of control spot with the same match ID. If this ratio is more than 1.5, then spotted protein was considered overexpressed, and a ratio of less than 0.5 indicates underexpressed protein spot. Unique protein spots are the spots that did not correlate in both the gels.

### In-Gel Digestion Protocol

After spots identification, the samples were taken for digestion. The band/spot was chopped and de-stained with 1:1 ratio of 15 mM potassium ferricyanide, K_3_ [Fe(CN)_6_], 50 mM Hypo for 15 min, and buffer washes and dehydration using ACN (acetonitrile). Sample preparation was done by 100 mM DTT at 56°C for 1 h and 250 mM IDA (iodoacetamide) at RT, in the dark for 45 min. Finally, samples were digested with trypsin at 37°C. The peptides were finally extracted using 0.1% TFA (trifluoroacetic acid), vacuum dried, and dissolved in 5 μl of TA buffer; 1.5 μl of the sample was mixed with 1.5 μl of HCCA (α-cyano-4-hydroxycinnamic acid) matrix, and 1.5 μl of the mixture was spotted onto the MALDI plate (Shevchenko et al., 2007).

### Matrix-Assisted Laser Desorption/Ionization-Time of Flight/Time of Flight Mass Spectrometry (MALDI-TOF/TOF MS)

The peptide mixture obtained was mixed in 1:1 ratio with 5 mg/ml HCAA in 1:2 ratio of 0.1% TFA and 100% ACN. Two microliters of sample was spotted onto the MALDI plate [(MTP 384 ground steel (Bruker Daltonics, Germany)]. The samples were analyzed on the MALDI-TOF/TOF ULTRAFLEX III instrument (Bruker Daltonics, Germany). FLEX ANALYSIS SOFTWARE (Version 3.3) was used to analyze the molecular weight of 500 laser shots with reflectron ion mode ranging between 500 and 5000 m/z for obtaining the MS-MS data. The masses obtained in the MS-MS were submitted for Mascot search in the “Viridiplantae” database to identify the protein (Koenig et al., 2008).

### Bioinformatics

CELLO V.2.5^1^ was used to predict subcellular localization, and predictions are based on two-level support vector machine (Wang et al., 2015). Gene ontology annotation of DEPs was conducted utilizing Protein Analysis through Evolutionary Relationships (PANTHER) Classification System version 16.0^2^ (Thomas et al., 2003; Mi et al., 2013). The identified DEPs were foreseen by WoLF PSORT^3^ UniprotKB^4^ and subjected to GeneMANIA database to identify protein interactions. The identified protein–protein interactions were constructed and visualized with Cytoscape (version 3.7.1) software. GeneMANIA finds alternative genes associated with collecting input genes, employing an extensive set of functional association data. We constructed a network using protein, genetic interactions, co-expression, co-localization, and protein domain similarity conditions. Modularity is used to identify sub-networks of interconnected nodes and aids in predicting the functionality (Agarwal et al., 2009).

### Quantitative RT-PCR (qRT-PCR)

Total RNA was extracted from the leaves of both plant varieties using RNAaqueous-Micro Total RNA isolation kit (Thermo Scientific, United States). NanoDrop One UV–Vis Spectrophotometer was used to check the concentration and purity of RNA (Thermo Scientific, United States). One microgram of total RNA was treated with DNAse I and the treated sample was used to synthesize cDNA with Revert PrimeScript cDNA synthesis kit (Takara Bio, Japan). The expression of mannitol-induced stress-responsive transcripts was analyzed by qRT-PCR using Applied Biosystems 7,500 Fast Real-Time PCR System. The following program was used: one cycle of 95°C for 30 s; 40 cycles of 95°C for 5 s and 60°C for 30 s. The qRT-PCR reactions (10 μl) included 30 ng of cDNA in 1 μl, TB Green Premix Ex Taq II (TliRNaseH Plus) (1×), PCR Forward Primer (10 μM) 1 μl, PCR Reverse Primer (10 μM) 1 μl, ROX Reference Dye (50×) 0.2 μl, and sterile purified water 1.8 μl.

## Data Availability Statement

The datasets presented in this study can be found in online repositories. The names of the repository/repositories and accession number(s) can be found below: https://www.ncbi.nlm.nih.gov/; YP_913194; Q3E956; XP_002876351; XP_002878640; NP_001314374; XP_015648830; Q7X9A0; YP_913194.

## Author Contributions

SN, RG, and SS conceived the idea and provided critical inputs to the concept. SN and SS planned the experiment. SN, SM, MA, JE, and SS contributed to analyze, interpret data and wrote the manuscript. All authors contributed to revision and approved the final version of the manuscript.

## Conflict of Interest

The authors declare that the research was conducted in the absence of any commercial or financial relationships that could be construed as a potential conflict of interest.

## Footnotes

1 http://cello.life.nctu.edu.tw

2 http://www.pantherdb.org

3 http://wolfpsort.org/

4 http://www.uniprot.org/

## References

[B1] Aasamaa K. Sõber A. (2011). Responses of stomatal conductance to simultaneous changes in two environmental factors. *Tree Physiol.* 31 855–864. 10.1093/treephys/tpr078 21856657

[B2] Agarwal P. Reddy M. K. Sopory S. K. Agarwal P. K. (2009). Plant rabs: characterization, functional diversity, and role in stress tolerance. *Plant Mol. Biol. Rep.* 27 417–430. 10.1007/s11105-009-0100-9

[B3] Ali F. Ahsan M. Ali Q. Kanwal N. (2017). Phenotypic stability of *Zea mays* grain yield and its attributing traits under drought stress. *Front. Plant Sci.* 8:1397. 10.3389/fpls.2017.01397 28878785 PMC5572255

[B4] Aranjuelo I. Molero G. Erice G. Avice J. C. Nogués S. (2011). Plant physiology and proteomics reveals the leaf response to drought in alfalfa (*Medicago sativa* L.). *J. Exp. Bot.* 62 111–123. 10.1093/jxb/erq249 20797998 PMC2993905

[B5] Ashraf M. Foolad M. R. (2007). Roles of glycine betaine and proline in improving plant abiotic stress resistance. *Environ. Exp. Bot.* 59 206–216. 10.1016/j.envexpbot.2005.12.006

[B6] Babar M. Saranga Y. Iqbal Z. Arif M. Zafar Y. (2009). Identification of QTLs and impact of selection from various environments (dry vs irrigated) on the genetic relationships among the selected cotton lines from f 6. *Afr. J.* 8 4802–4810.

[B7] Banerjee A. Roychoudhury A. (2015). WRKY proteins: signaling and regulation of expression during abiotic stress responses. *Sci. World J.* 2015 807560. 10.1155/2015/807560 25879071 PMC4387944

[B8] Barrs H. Weatherley P. (1962). A re-examination of the relative turgidity technique for estimating water deficits in leaves. *Aust. J. Biol. Sci.* 15 413–428. 10.1071/bi9620413

[B9] Bates L. S. Waldren R. P. Teare I. D. (1973). Rapid determination of free proline for water – stress studies. *Plant Soil* 207 205–207. 10.1007/bf00018060

[B10] Becana M. Dalton D. A. Moran J. F. Iturbe-Ormaetxe I. Matamoros M. A. Rubio M. C. (2000). Reactive oxygen species and antioxidants in legume nodules. *Physiol. Plant.* 109 372–381. 10.1034/j.1399-3054.2000.100402.x 11841302

[B11] Bhatt R. M. Rao N. S. (2005). Influence of pod load on response of okra to water stress. *Ind. J. Plant Physiol.* 10 54–59.

[B12] Bourne H. R. Sanders D. A. McCormick F. (1990). The GTPase superfamily: a conserved switch for diverse cell functions. *Nature* 348 125–132. 10.1038/348125a0 2122258

[B13] Brandão A. R. Barbosa H. S. Arruda M. A. Z. (2010). Image analysis of two-dimensional gel electrophoresis for comparative proteomics of transgenic and non-transgenic soybean seeds. *J. Proteomics* 73 1433–1440. 10.1016/j.jprot.2010.01.009 20123049

[B14] Cao Y. Luo Q. Tian Y. Meng F. (2017). Physiological and proteomic analyses of the drought stress response in *Amygdalus mira* (Koehne) Yü et Lu roots. *BMC Plant Biol.* 17:53. 10.1186/s12870-017-1000-z 28241796 PMC5327565

[B15] Chai W. Peng X. Liu B. Wang J. Zhu Z. Liu Y. (2018). Comparative genomics, whole-genome re-sequencing and expression profile analysis of nucleobase:Cation symporter 2 (NCS2) genes in maize. *Front. Plant Sci.* 9:856. 10.3389/fpls.2018.00856 30002663 PMC6031955

[B16] Chen Z. Xu J. Wang F. Wang L. Xu Z. (2019). Morpho-physiological and proteomic responses to water stress in two contrasting tobacco varieties. *Sci. Rep.* 9:12188. 10.1038/s41598-019-54995-1 31811189 PMC6898209

[B17] Cohen D. Bogeat-Triboulot M. B. Tisserant E. Balzergue S. Martin-Magniette M. L. Lelandais G. (2010). Comparative transcriptomics of drought responses in *Populus*: a meta-analysis of genome-wide expression profiling in mature leaves and root apices across two genotypes. *BMC Genomics* 11:630. 10.1186/1471-2164-11-630 21073700 PMC3091765

[B18] Conner A. J. Jacobs J. M. E. (1999). Genetic engineering of crops as potential source of genetic hazard in the human diet. *Mutat. Res. Genet. Toxicol. Environ. Mutagen.* 443 223–234. 10.1016/S1383-5742(99)00020-4 10415441

[B19] Cramer G. R. van Sluyter S. C. Hopper D. W. Pascovici D. Keighley T. Haynes P. A. (2013). Proteomic analysis indicates massive changes in metabolism prior to the inhibition of growth and photosynthesis of grapevine (*Vitis vinifera* L.) in response to water deficit. *BMC Plant Biol.* 13:49. 10.1186/1471-2229-13-49 23514573 PMC3608200

[B20] Deeba F. Pandey A. K. Ranjan S. Mishra A. Singh R. Sharma Y. K. (2012). Physiological and proteomic responses of cotton (*Gossypium herbaceum* L.) to drought stress. *Plant Physiol. Biochem.* 53 6–18. 10.1016/j.plaphy.2012.01.002 22285410

[B21] Dubouzet J. G. Sakuma Y. Ito Y. Kasuga M. Dubouzet E. G. Miura S. (2003). OsDREB genes in rice, *Oryza sativa* L., encode transcription activators that function in drought-, high-salt- and cold-responsive gene expression. *Plant J.* 33 751–763. 10.1046/j.1365-313X.2003.01661.x 12609047

[B22] Gong C. Y. Li Q. Yu H. T. Wang Z. Wang T. (2012). Proteomics insight into the biological safety of transgenic modification of rice as compared with conventional genetic breeding and spontaneous genotypic variation. *J. Proteome Res.* 11 3019–3029. 10.1021/pr300148w 22509807

[B23] Gong C. Y. Wang T. (2013). Proteomic evaluation of genetically modified crops: current status and challenges. *Front. Plant Sci.* 4:41. 10.3389/fpls.2013.00041 23471542 PMC3590489

[B24] Greenbaum D. Colangelo C. Williams K. Gerstein M. (2003). Comparing protein abundance and mRNA expression levels on a genomic scale. *Genome Biol.* 4:117. 10.1186/gb-2003-4-9-117 PMC193646 12952525

[B25] Gujarathi B. G. Hegde B. A. Patil T. M. (1984). Proline efficiency of photosynthetic carboxylation and enzyme activity in sorghum genotype and peanut under water stress. *Adv. Photosynth. Res.* 4 399–402. 10.1007/978-94-017-6368-4

[B26] Haake V. Cook D. Riechmann J. L. Pineda O. Thomashow M. F. Zhang J. Z. (2002). Transcription factor CBF4 is a regulator of drought adaptation in *Arabidopsis*. *Plant Physiol.* 130 639–648. 10.1104/pp.006478 12376631 PMC166593

[B27] Hare P. D. Cress W. A. van Staden J. (1999). Proline synthesis and degradation: a model system for elucidating stress-related signal transduction. *J. Exp. Bot.* 50 413–434. 10.1093/jxb/50.333.413 12432039

[B28] Hasanuzzaman M. Nahar K. Alam M. M. Roychowdhury R. Fujita M. (2013). Physiological, biochemical, and molecular mechanisms of heat stress tolerance in plants. *Int. J. Mol. Sci.* 14 9643–9684. 10.3390/ijms14059643 23644891 PMC3676804

[B29] Jiang Y. Yang B. Harris N. S. Deyholos M. K. (2007). Comparative proteomic analysis of NaCl stress-responsive proteins in *Arabidopsis* roots. *J. Exp. Bot.* 58 3591–3607. 10.1093/jxb/erm207 17916636

[B30] Jin L. Huang B. Li H. Liu J. (2009). Expression profiles and transactivation analysis of a novel ethylene-responsive transcription factor gene GhERF5 from cotton. *Prog. Nat. Sci.* 19 563–572. 10.1016/j.pnsc.2008.05.036

[B31] Kantar M. Lucas S. J. Budak H. (2011). “Drought stress: molecular genetics and genomics approaches,” in *Advances in Botanical Research*, 1st Edn, ed. Turkan I. (Burlington, MA: Elsevier Ltd). 10.1016/B978-0-12-387692-8.00013-8

[B32] Kaur G. Asthir B. (2015). Proline: a key player in plant abiotic stress tolerance. *Biol. Plant.* 59 609–619. 10.1007/s10535-015-0549-3

[B33] Kim Y. M. Han Y. J. Hwang O. J. Lee S. S. Shin A. Y. Kim S. Y. (2012). Overexpression of *Arabidopsis* translationally controlled tumor protein gene AtTCTP enhances drought tolerance with rapid ABA-induced stomatal closure. *Mol. Cells* 33 617–626. 10.1007/s10059-012-0080-8 22610367 PMC3887759

[B34] Koenig T. Menze B. H. Kirchner M. Monigatti F. Parker K. C. Patterson T. (2008). Robust prediction of the MASCOT score for an improved quality assessment in mass spectrometric proteomics. *J. Proteome Res.* 7 3708–3717. 10.1021/pr700859x 18707158

[B35] Koroleva O. A. Tomlinson M. L. Leader D. Shaw P. Doonan J. H. (2005). High-throughput protein localization in *Arabidopsis* using Agrobacterium-mediated transient expression of GFP-ORF fusions. *Plant J.* 41 162–174. 10.1111/j.1365-313X.2004.02281.x 15610358

[B36] Kottapalli K. R. Payton P. Rakwal R. Agrawal G. K. Shibato J. Burow M. (2008). Proteomics analysis of mature seed of four peanut cultivars using two-dimensional gel electrophoresis reveals distinct differential expression of storage, anti-nutritional, and allergenic proteins. *Plant Sci.* 175 321–329. 10.1016/j.plantsci.2008.05.005

[B37] Law R. D. Crafts-Brandner S. J. Salvucci M. E. (2001). Heat stress induces the synthesis of a new form of ribulose-1,5-bisphosphate carboxylase/oxygenase activase in cotton leaves. *Planta* 214 117–125. 10.1007/s004250100592 11762161

[B38] Lawlor D. W. Cornic G. (2002). Photosynthetic carbon assimilation and associated PHOTOSYNTHESIS UNDER. *Regulation* 44 275–294. 10.1046/j.0016-8025.2001.00814.x 11841670

[B39] Lichtenthaler H. K. Wellburn A. R. (1983). Determinations of total carotenoids and chlorophylls a and b of leaf extracts in different solvents. *Biochem. Soc. Trans.* 11 591–592. 10.1042/bst0110591

[B40] Liu C. Zhang T. (2017). Expansion and stress responses of the AP2/EREBP superfamily in cotton. *BMC Genomics* 18:118. 10.1186/s12864-017-3517-9 28143399 PMC5282909

[B41] Lloyd C. W. (1989). The plant cytoskeleton. *Curr. Opin. Cell Biol.* 1 30–35. 10.1016/S0955-0674(89)80033-X 2698207

[B42] Lubbers E. L. Chee P. W. Saranga Y. Paterson A. H. (2007). “Recent advances and future prospective in molecular breeding of cotton for drought and salinity stress tolerance,” in *Advances in Molecular Breeding Toward Drought and Salt Tolerant Crops*, eds Jenks M. A. Hasegawa P. M. Jain S. M. (Dordrecht: Springer), 775–796. 10.1007/978-1-4020-5578-2_31

[B43] Mahmood A. Haider M. S. Ali Q. Nasir I. A. (2017). Multivariate analysis to assess abscisic acid content association with different physiological and plant growth related traits of *Petunia*. *Acta Agric. Slov.* 109 165–173. 10.14720/aas.2017.109.2.02

[B44] Man D. Bao Y. X. Han L. B. Zhang X. (2011). Drought tolerance associated with proline and hormone metabolism in two tall fescue cultivars. *HortScience* 46 1027–1032. 10.21273/hortsci.46.7.1027

[B45] Mi H. Muruganujan A. Thomas P. D. (2013). PANTHER in 2013: modeling the evolution of gene function, and other gene attributes, in the context of phylogenetic trees. *Nucleic Acids Res.* 41 377–386. 10.1093/nar/gks1118 23193289 PMC3531194

[B46] Mostafavi S. Ray D. Warde-Farley D. Grouios C. Morris Q. (2008). GeneMANIA: a real-time multiple association network integration algorithm for predicting gene function. *Genome Biol.* 9(Suppl. 1):S4. 10.1186/gb-2008-9-s1-s4 18613948 PMC2447538

[B47] Możdżeń K. Bojarski B. Rut G. Migdałek G. Repka P. Rzepka A. (2015). Effect of drought stress induced by mannitol on physiological parameters of maize (*Zea mays* L.) seedlings and plants. *J. Microbiol. Biotechnol. Food Sci.* 04 86–91. 10.15414/jmbfs.2015.4.special2.86-91

[B48] Nakano Y. Asada K. (1981). Hydrogen peroxide is scavenged by ascorbate specific peroxidase in spinach chloroplasts. *Plant Cell Physiol.* 22 867–880.

[B49] Nakashima K. Takasaki H. Mizoi J. Shinozaki K. Yamaguchi-Shinozaki K. (2012). NAC transcription factors in plant abiotic stress responses. *Biochim. Biophys. Acta* 1819 97–103. 10.1016/j.bbagrm.2011.10.005 22037288

[B50] O’Farrell P. H. (1975). High resolution of proteins ^∗^ electrophoresis. *J. Biol. Chem.* 250 4007–4021. 10.1016/j.bbi.2008.05.010 236308 PMC2874754

[B51] Olsen A. N. Ernst H. A. Lo Leggio L. Skriver K. (2005). NAC transcription factors: structurally distinct, functionally diverse. *Trends Plant Sci.* 10 79–87. 10.1016/j.tplants.2004.12.010 15708345

[B52] Parida A. K. Dagaonkar V. S. Phalak M. S. Umalkar G. V. Aurangabadkar L. P. (2007). Alterations in photosynthetic pigments, protein and osmotic components in cotton genotypes subjected to short-term drought stress followed by recovery. *Plant Biotechnol. Rep.* 1 37–48. 10.1007/s11816-006-0004-1

[B53] Per T. S. Khan M. I. R. Anjum N. A. Masood A. Hussain S. J. Khan N. A. (2018). Jasmonates in plants under abiotic stresses: crosstalk with other phytohormones matters. *Environ. Exp. Bot.* 145 104–120. 10.1016/j.envexpbot.2017.11.004

[B54] Pérez-Pérez J. M. Esteve-Bruna D. González-Bayón R. Kangasjärvi S. Caldana C. Hannah M. A. (2013). Functional redundancy and divergence within the *Arabidopsis* RETICULATA-RELATED gene family. *Plant Physiol.* 162 589–603. 10.1104/pp.113.217323 23596191 PMC3668055

[B55] Qiao Z. X. Huang B. Liu J. Y. (2008). Molecular cloning and functional analysis of an ERF gene from cotton (*Gossypium hirsutum*). *Biochim. Biophys. Acta* 1779 122–127. 10.1016/j.bbagrm.2007.10.003 18078841

[B56] Ren Y. Lv J. Wang H. Li L. Peng Y. Qu L. J. (2009). A comparative proteomics approach to detect unintended effects in transgenic *Arabidopsis*. *J. Genet. Genomics* 36 629–639. 10.1016/S1673-8527(08)60155-1 19840761

[B57] Sánchez F. J. Manzanares M. De Andres E. F. Tenorio J. L. Ayerbe L. (1998). Turgor maintenance, osmotic adjustment and soluble sugar and proline accumulation in 49 pea cultivars in response to water stress. *Field Crop. Res.* 59 225–235. 10.1016/S0378-4290(98)00125-7

[B58] Sánchez-Blanco M. J. Rodríguez P. Morales M. A. Ortuo M. F. Torrecillas A. (2002). Comparative growth and water relations of *Cistus albidus* and *Cistus monspeliensis* plants during water deficit conditions and recovery. *Plant Sci.* 162 107–113. 10.1016/S0168-9452(01)00540-4

[B59] Seki M. Umezawa T. Urano K. Shinozaki K. (2007). Regulatory metabolic networks in drought stress responses. *Curr. Opin. Plant Biol.* 10 296–302. 10.1016/j.pbi.2007.04.014 17468040

[B60] Selote D. S. Khanna-Chopra R. (2004). Drought-induced spikelet sterility is associated with an inefficient antioxidant defence in rice panicles. *Physiol. Plant.* 121 462–471. 10.1111/j.1399-3054.2004.00341.x

[B61] Seo Y. J. Park J. B. Cho Y. J. Jung C. Seo H. S. Park S. K. (2010). Overexpression of the ethylene-responsive factor gene BrERF4 from *Brassica rapa* increases tolerance to salt and drought in *Arabidopsis* plants. *Mol. Cells* 30 271–277. 10.1007/s10059-010-0114-z 20803085

[B62] Serrato A. J. Pérez-Ruiz J. M. Spínola M. C. Cejudo F. J. (2004). A novel NADPH thioredoxin reductase, localised in the chloroplast, which deficiency causes hypersensitivity to abiotic stress in *Arabidopsis thaliana*. *J. Biol. Chem.* 279 43821–43827. 10.1074/jbc.M404696200 15292215

[B63] Shah K. Kumar R. G. Verma S. Dubey R. S. (2001). Effect of cadmium on lipid peroxidation, superoxide anion generation and activities of antioxidant enzymes in growing rice seedlings. *Plant Sci.* 161 1135–1144. 10.1016/S0168-9452(01)00517-9

[B64] Shao H. B. Chu L. Y. Jaleel C. A. Zhao C. X. (2008). Water-deficit stress-induced anatomical changes in higher plants. *C. R. Biol.* 331 215–225. 10.1016/j.crvi.2008.01.002 18280987

[B65] Shao S. Brown A. Santhanam B. Hegde R. S. (2015). Structure and assembly pathway of the ribosome quality control complex. *Mol. Cell* 57 433–444. 10.1016/j.molcel.2014.12.015 25578875 PMC4321881

[B66] Shevchenko A. Tomas H. Havliš J. Olsen J. V. Mann M. (2007). In-gel digestion for mass spectrometric characterization of proteins and proteomes. *Nat. Protoc.* 1 2856–2860. 10.1038/nprot.2006.468 17406544

[B67] Sobhanian H. Razavizadeh R. Nanjo Y. Ehsanpour A. A. Jazii F. R. Motamed N. (2010). Proteome analysis of soybean leaves, hypocotyls and roots under salt stress. *Proteome Sci.* 8:19. 10.1186/1477-5956-8-19 20350314 PMC2859372

[B68] Spreitzer R. J. (2003). Role of the small subunit in ribulose-1,5-bisphosphate carboxylase/oxygenase. *Arch. Biochem. Biophys.* 414 141–149. 10.1016/S0003-9861(03)00171-1 12781765

[B69] Sultan M. A. R. F. Hui L. Yang L. J. Xian Z. H. (2012). Assessment of drought tolerance of some triticum l. species through physiological indices. *Czech J. Genet. Plant Breed.* 48 178–184. 10.17221/21/2012-cjgpb

[B70] Sun T. Jia D. Huang L. Shao Y. Ma F. (2016). Comprehensive genomic identification and expression analysis of the nucleobase-ascorbate transporter (NAT) gene family in apple. *Sci. Hortic.* 198 473–481. 10.1016/j.scienta.2015.09.034

[B71] Szabados L. Savouré A. (2010). Proline: a multifunctional amino acid. *Trends Plant Sci.* 15 89–97. 10.1016/j.tplants.2009.11.009 20036181

[B72] Tan Y. Yi X. Wang L. Peng C. Sun Y. Wang D. (2016). Comparative proteomics of leaves from phytase-transgenic maize and its non-transgenic isogenic variety. *Front. Plant Sci.* 7:1211. 10.3389/fpls.2016.01211 27582747 PMC4987384

[B73] Tezara W. Mitchell V. J. Driscoll S. D. Lawlor D. W. (1999). Water stress inhibits plant photosynthesis by decreasing coupling factor and ATP. *Nature* 401 914–917. 10.1038/44842

[B74] Thomas P. D. Campbell M. J. Kejariwal A. Mi H. Karlak B. Daverman R. (2003). PANTHER: a library of protein families and subfamilies indexed by function. *Genome Res.* 13 2129–2141. 10.1101/gr.772403 12952881 PMC403709

[B75] Tran L. S. P. Nishiyama R. Yamaguchi-Shinozaki K. Shinozaki K. (2010). Potential utilization of NAC transcription factors to enhance abiotic stress tolerance in plants by biotechnological approach. *GM Crops* 1 32–39. 10.4161/gmcr.1.1.10569 21912210

[B76] Ullah F. Bano A. Nosheen A. (2012). Effects of plant growth regulators on growth and oil quality of canola (*Brassica napus* L.) under drought stress. *Pak. J. Bot.* 44 1873–1880.

[B77] Valero-Galván J. González-Fernández R. Navarro-Cerrillo R. M. Gil-Pelegrín E. Jorrín-Novo J. V. (2013). Physiological and proteomic analyses of drought stress response in Holm oak provenances. *J. Proteome Res.* 12 5110–5123. 10.1021/pr400591n 24088139

[B78] van Rossum M. W. P. C. van der Plas L. H. W. (1997). Oxygen stress in tulip bulb scale micropropagation. *Phyton* 37 291–296.

[B79] Venugopalan M. V. Sankaranarayanan K. Blaise D. Nalayini P. Prahraj C. S. Bandla G. (2009). Bt cotton (*Gossypium* sp.) in India and its agronomic requirements – a review. *Ind. J. Agron.* 54 343–360.

[B80] Wang F. X. Ma Y. P. Yang C. L. Zhao P. M. Yao Y. Jian G. L. (2011). Proteomic analysis of the sea-island cotton roots infected by wilt pathogen *Verticillium dahliae*. *Proteomics* 11 4296–4309. 10.1002/pmic.201100062 21928292

[B81] Wang L. Wang X. Jin X. Jia R. Huang Q. Tan Y. (2015). Comparative proteomics of Bt-transgenic and non-transgenic cotton leaves. *Proteome Sci.* 13:15. 10.1186/s12953-015-0071-8 25949214 PMC4422549

[B82] Wang W. Vinocur B. Altman A. (2003). Plant responses to drought, salinity and extreme temperatures: towards genetic engineering for stress tolerance. *Planta* 218 1–14. 10.1007/s00425-003-1105-5 14513379

[B83] Wang X. Cai X. Xu C. Wang Q. Dai S. (2016). Drought-responsive mechanisms in plant leaves revealed by proteomics. *Int. J. Mol. Sci.* 17:1706. 10.3390/ijms17101706 27763546 PMC5085738

[B84] Wang X. Li X. Li Y. (2007). A modified Coomassie Brilliant Blue staining method at nanogram sensitivity compatible with proteomic analysis. *Biotechnol. Lett.* 29 1599–1603. 10.1007/s10529-007-9425-3 17563857

[B85] Wilkins T. A. Rajasekaran K. Anderson D. M. (2000). Cotton biotechnology. *Crit. Rev. Plant Sci.* 19 511–550. 10.1080/07352680091139286

[B86] Xie H. Yang D. H. Yao H. Bai G. Zhang Y. H. Xiao B. G. (2016). ITRAQ-based quantitative proteomic analysis reveals proteomic changes in leaves of cultivated tobacco (*Nicotiana tabacum*) in response to drought stress. *Biochem. Biophys. Res. Commun.* 469 768–775. 10.1016/j.bbrc.2015.11.133 26692494

[B87] Xu Y. H. Liu R. Yan L. Liu Z. Q. Jiang S. C. Shen Y. Y. (2012). Light-harvesting chlorophyll a/b-binding proteins are required for stomatal response to abscisic acid in *Arabidopsis*. *J. Exp. Bot.* 63 1095–1106. 10.1093/jxb/err315 22143917 PMC3276081

[B88] Yang Y. W. Bian S. M. Yao Y. Liu J. Y. (2008). Comparative proteomic analysis provides new insights into the fiber elongating process in cotton. *J. Proteome Res.* 7 4623–4637. 10.1021/pr800550q 18823139

[B89] Zhang H. B. Li Y. Wang B. Chee P. W. (2008). Recent advances in cotton genomics. *Int. J. Plant Genomics* 2008:742304. 10.1155/2008/742304 18288253 PMC2233810

[B90] Zhang N. Zhang L. Zhao L. Ren Y. Cui D. Chen J. (2017). iTRAQ and virus-induced gene silencing revealed three proteins involved in cold response in bread wheat. *Sci. Rep.* 7:7524. 10.1038/s41598-017-08069-9 28790462 PMC5548720

[B91] Zhang Q. Rami Alfarra M. Worsnop D. R. Allan J. D. Coe H. Canagaratna M. R. (2005). Deconvolution and quantification of hydrocarbon-like and oxygenated organic aerosols based on aerosol mass spectrometry. *Environ. Sci. Technol.* 39 4938–4952. 10.1021/es048568l 16053095

[B92] Zhou S. Li M. Guan Q. Liu F. Zhang S. Chen W. (2015). Physiological and proteome analysis suggest critical roles for the photosynthetic system for high water-use efficiency under drought stress in *Malus*. *Plant Sci.* 236 44–60. 10.1016/j.plantsci.2015.03.017 26025520

[B93] Zhu Y. Wang B. Tang K. Hsu C. C. Xie S. Du H. (2017). An *Arabidopsis* nucleoporin NUP85 modulates plant responses to ABA and salt stress. *PLoS Genet.* 13:e1007124. 10.1371/journal.pgen.1007124 29232718 PMC5741264

[B94] Zou J. J. Wei F. J. Wang C. Wu J. J. Ratnasekera D. Liu W. X. (2010). *Arabidopsis* calcium-dependent protein kinase cpk10 functions in abscisic acid- and Ca2+-mediated stomatal regulation in response to drought stress. *Plant Physiol.* 154 1232–1243. 10.1104/pp.110.157545 20805328 PMC2971602

