# Supplementary material for: Morpho-Physiological and Proteomic Response of Bt-Cotton and Non-Bt Cotton to Drought Stress
Source: Front Plant Sci. 2021 May 10;12:663576. doi: 10.3389/fpls.2021.663576 (PMC8143030; doi:10.3389/fpls.2021.663576)

**Morpho-physiological and proteomic response of Bt-cotton and Non-Bt cotton to drought stress**

Swetha Sudha Nagamalla^a^, AlaparthiMaliniDevi^a^, Sunitha Mellacheruvu^a^, GundetiRavindar^a^, Earrawandla Jana Priya Sony^a^ and Someswar Rao Sagurthi^a*^

^a^Molecular Medicine Lab, Department of Genetics & Biotechnology, Osmania University, Hyderabad, Telangana-500007, India


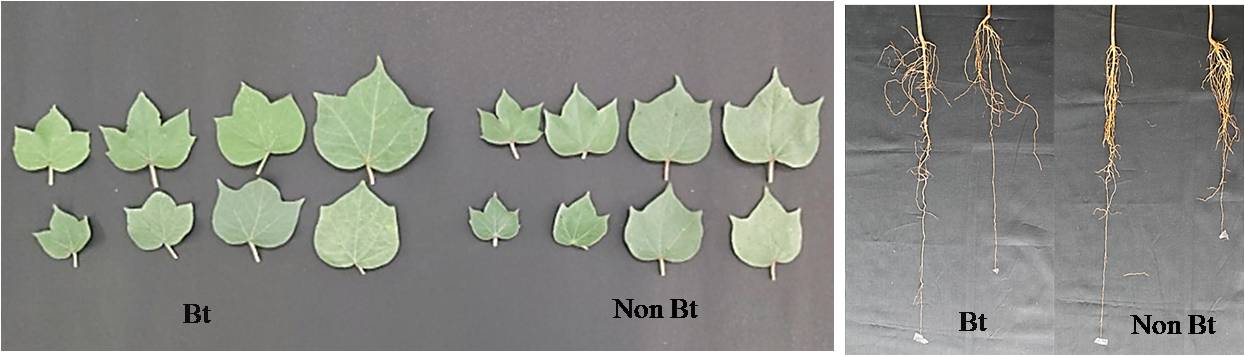


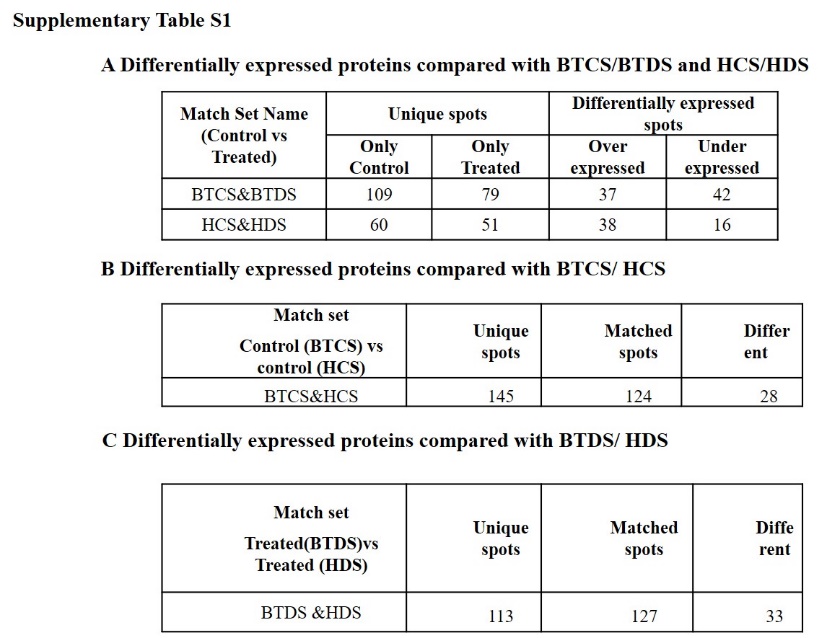

Supplement: Supplementary file 1 [file Data_Sheet_1.docx]
